# When Macrophages Heal and When They Scar: Timing in Corneal Fibrosis

**DOI:** 10.3390/life16071090

**Published:** 2026-06-29

**Authors:** Amal Yaghmour, Zohreh Arabpour, Hannah Al-Khudari, Ali Djalilian

**Affiliations:** Department of Ophthalmology and Visual Science, University of Illinois, Chicago, IL 60612, USA; ayaghm3@uic.edu (A.Y.); arabpour@uic.edu (Z.A.); hannahalkhudari@gmail.com (H.A.-K.)

**Keywords:** corneal fibrosis, macrophage polarization, timing-based immunotherapy

## Abstract

Corneal fibrosis and scarring remain leading causes of vision impairment and blindness globally, particularly following trauma, infection, or surgical intervention. Macrophages, as central mediators of immune and repair responses, orchestrate key phases of corneal wound healing. Their functional states, ranging from pro-inflammatory to pro-resolving, are tightly regulated by environmental cues and timing. Following corneal injury, recruited macrophages undergo temporally distinct activation programs. Early inflammatory macrophage responses support debris clearance, pathogen defense, and initiation of stromal repair, whereas persistence of inflammatory and profibrotic macrophage signaling beyond the acute healing phase is associated with sustained TGF-β activity, fibroblast-to-myofibroblast differentiation, excessive extracellular matrix deposition, and stromal haze formation. Conversely, timely transition toward pro-resolving macrophage states promotes inflammation resolution, myofibroblast clearance, and restoration of corneal transparency. Recent single-cell transcriptomic profiling reveals substantial heterogeneity among macrophage populations in the cornea, highlighting the limitations of the oversimplified M1/M2 classification. This review aims to define how macrophage heterogeneity and temporal dynamics regulate corneal wound healing outcomes, with particular emphasis on the balance between fibrosis and regeneration, and to provide a conceptual framework relevant to both basic researchers and translational clinicians.

## 1. Introduction

The cornea plays a critical role in vision as the primary refractive surface of the eye. This highly specialized, multilayered tissue is composed of precisely organized epithelial, stromal, and endothelial layers, each contributing to light refraction, mechanical stability, and protection of the ocular surface [1]. The precise spatial arrangement of corneal cells and extracellular matrix (ECM) components is essential for maintaining optical transparency and ensuring accurate visual signal transmission [2,3]. In addition to its refractive function, the cornea serves as a physical and biochemical barrier, protecting the eye from environmental pathogens [4].

A defining feature of the cornea is its avascularity, which is essential for maintaining optical clarity by minimizing light scattering and preserving immune privilege [5]. The absence of blood and lymphatic vessels permits efficient light transmission to the retina and minimizes light scattering that would otherwise impair vision. This immune-privileged status is actively sustained by tightly regulated cellular and molecular mechanisms that balance immune surveillance with controlled inflammatory responses [6]. However, disruption of this finely tuned equilibrium during injury or disease can lead to dysregulated wound-healing responses, making the cornea more susceptible to chronic inflammation and fibrosis [7].

Damage to the cornea can lead to profound visual impairment or blindness. Globally, approximately 253 million individuals are affected by blindness or visual impairment, with an estimated 6.17 million cases (2.4%) associated with corneal diseases, including infectious keratitis, traumatic injury, degenerative disorders, and chronic inflammatory conditions [8]. Corneal blindness thus represents a substantial public health burden and remains a leading cause of preventable vision loss, particularly in low- and middle-income countries, where access to timely diagnosis and effective treatment is limited [9].

Although considerable progress has been made in pharmacological and surgical management of corneal disorders, existing treatments often fail to achieve durable clinical outcomes or prevent disease progression. This limitation is especially apparent in conditions marked by persistent inflammation, aberrant stromal remodeling, fibrosis, and limbal stem cell deficiency [10]. Conventional approaches such as topical anti-inflammatory or immunosuppressive therapies may alleviate acute symptoms, yet they rarely correct the underlying disturbances in wound-healing pathways that ultimately lead to chronic scarring and loss of corneal transparency [10,11]. For advanced disease, corneal transplantation remains the definitive therapeutic option; however, its clinical utility is constrained by donor tissue scarcity, immune-mediated rejection, graft failure, and postoperative complications [11]. Collectively, these challenges highlight the need for novel therapeutic strategies that support true tissue regeneration while maintaining corneal immune balance.

Mesenchymal stromal cells (MSCs) have gained considerable attention as a promising therapeutic strategy in ophthalmic research. MSCs are characterized by broad immunomodulatory, anti-inflammatory, anti-fibrotic, and regenerative capacities and have demonstrated encouraging efficacy in preclinical studies as well as early clinical investigations targeting ocular surface diseases [12,13]. Importantly, growing evidence indicates that MSCs exert their therapeutic effects primarily by reshaping the local tissue microenvironment, thereby regulating immune activity, ECM remodeling, and endogenous repair processes [14].

Consistent with this concept, the beneficial effects of MSC-based therapies are now widely recognized to be mediated largely through paracrine signaling rather than long-term engraftment or direct differentiation [15,16]. Among these paracrine mediators are extracellular vesicles (EVs) secreted by MSCs have emerged as critical effectors of tissue repair and immunoregulation [14]. MSC-derived EVs carry a diverse cargo of bioactive molecules, including proteins, lipids, messenger RNAs, and microRNAs, which can profoundly influence recipient cell behavior [17,18]. Recent advances in ocular exosome research have further highlighted their potential as therapeutic delivery vehicles owing to their intrinsic biocompatibility, low immunogenicity, ability to traverse biological barriers, and capacity for engineering through cargo modification, surface functionalization, and hybrid vesicle design. Experimental studies have demonstrated exosome-mediated neuroprotection, anti-angiogenic delivery, and targeted ocular drug transport, underscoring the translational potential of EV-based therapies for corneal disease and regenerative ophthalmology [19]. In multiple disease models, these vesicles have been shown to modulate immune cell activation, suppress inflammatory responses, promote epithelial regeneration, and facilitate stromal remodeling [20,21]. Notably, EV-based approaches offer a cell-free therapeutic alternative that may overcome several safeties, logistical, and regulatory challenges associated with live cell transplantation [22].

Beyond their direct effects on corneal resident cells, MSC-based therapies exert profound immunoregulatory influences through the education and functional reprogramming of innate immune cells, particularly macrophages [23,24]. Macrophages are central regulators of corneal wound healing and display substantial functional plasticity, enabling them to either support tissue regeneration or drive fibrotic scarring depending on environmental cues and temporal context [7,25]. MSC-educated macrophages, often characterized by a shift toward anti-inflammatory and pro-regenerative programs, play a pivotal role in restoring immune balance within the injured cornea [26]. These macrophages contribute to resolution of inflammation, suppression of excessive fibroblast activation, and promotion of organized ECM deposition through the release of anti-inflammatory cytokines, growth factors, and matrix-modulating enzymes [27].

Emerging evidence further indicates that MSC-derived EVs are key mediators of macrophage reprogramming, transferring bioactive molecular cargo that alters macrophage metabolism, gene expression, and functional behavior [28,29].

Importantly, accumulating evidence indicates that macrophage-driven outcomes in the cornea are determined not simply by macrophage presence or phenotype, but by the timing and persistence of specific macrophage programs during wound healing [30,31]. In a murine corneal alkali-burn model, Lamy et al. demonstrated a sequential transition of recruited mononuclear phagocytes, with Ly6C^hi^ inflammatory cells predominating at day 1 post-injury, Ly6C^int^ transitional populations emerging by day 3, and Ly6C^neg^ reparative populations becoming dominant by day 6, highlighting the dynamic nature of macrophage responses during corneal repair [32]. Consistent with these observations, successful corneal healing requires timely resolution of the initial inflammatory phase and progression toward reparative macrophage programs. In contrast, prolonged macrophage activation and sustained production of profibrotic mediators, particularly TGF-β, promote fibroblast-to-myofibroblast differentiation, excessive extracellular matrix deposition, stromal haze formation, and fibrosis [11]. Thus, the duration of macrophage activation may be as important as macrophage phenotype in determining whether corneal wound healing culminates in regeneration or pathological scarring [11,33].

**Figure 1:**
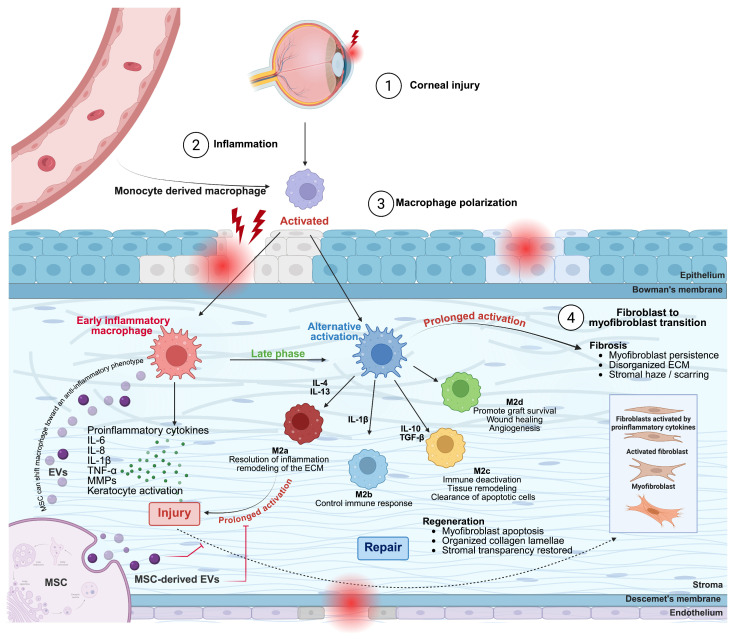
Macrophage polarization and stromal remodeling following corneal injury. Corneal injury (1) triggers an inflammatory response characterized by the recruitment of monocyte-derived macrophages (2). These macrophages become activated and undergo polarization (3) into distinct phenotypes depending on the local microenvironment. In the early inflammatory phase, classically activated (M1-like) macrophages secrete pro-inflammatory cytokines (e.g., IL-6, IL-8, IL-1β, TNF-α), matrix metalloproteinases (MMPs), and extracellular vesicles (EVs), promoting keratocyte activation and tissue injury. During the later phase, alternatively activated macrophages (M2 subtypes: M2a, M2b, and M2c) contribute to resolution of inflammation, extracellular matrix (ECM) remodeling, immune regulation, angiogenesis, and tissue repair through the release of cytokines such as IL-4, IL-13, IL-10, and TGF-β. Mesenchymal stromal cells (MSCs) and MSC-derived EVs further modulate macrophage activation and polarization. Prolonged or dysregulated macrophage activation leads to fibroblast-to-myofibroblast transition (4), resulting in fibrosis, stromal haze, disorganized ECM, and scarring. In contrast, timely resolution of inflammation promotes myofibroblast apoptosis, organized collagen lamellae, restoration of stromal transparency, and successful corneal repair. In the diagram, numbered circles indicate chronological stages of the pathway, arrows denote cellular transitions or signaling directions, and distinct colors are used to differentiate between specific cell phenotypes and injury/repair phases. Created in BioRender. Yaghmour, A. (2026) https://BioRender.com/5gmq5dd, accessed on 23 June 2026.

This review provides a comprehensive and up-to-date assessment of unmet clinical needs in the management of corneal disease and critically evaluates the therapeutic potential of MSC-based strategies. With particular emphasis the role of macrophages in corneal wound healing and fibrosis, with attention to the temporal dynamics that determine whether immune responses promote regeneration or drive pathological scarring. In addition, this review highlights nanovesicle-mediated mechanisms as an emerging, cell-free therapeutic approach that may overcome key limitations of conventional treatments and cell-based therapies. Overall, the central aim of this review is to integrate mechanistic insights into macrophage heterogeneity and timing with emerging immunomodulatory therapies, providing a conceptual framework for basic scientists and translational clinicians seeking to prevent corneal fibrosis while preserving tissue transparency. The sequence of macrophage activation, stromal remodeling, and the balance between regenerative healing and fibrosis is summarized in Figure 1.

## 2. Composition of the Cornea

Corneal transparency is essential for maintaining the eye’s refractive power and high visual acuity. This property depends on the cornea’s highly specialized structural organization, precise cellular composition, avascularity, and tightly regulated immune environment [1,2]. Disruption of these features, particularly during injury, alters immune cell behavior and stromal remodeling, predisposing the tissue to chronic inflammation, fibrosis, and loss of optical clarity. Understanding corneal structure in the context of immune regulation is therefore critical for elucidating how macrophage responses shape regenerative versus fibrotic healing outcomes.

### 2.1. Structural Organization of the Cornea

#### 2.1.1. Corneal Epithelium

The corneal epithelium forms the primary barrier between the ocular surface and the external environment and is a critical regulator of early immune signaling following injury. Beyond its role in maintaining refractive precision and limiting microbial invasion, epithelial cells actively participate in wound healing by releasing cytokines and chemokines, including interleukin-1β (IL-1β), interleukin-6 (IL-6), interleukin-8 (IL-8), and tumor necrosis factor-α (TNF-α) [34,35]. These mediators initiate inflammatory cascades that recruit and activate immune cells within the underlying stroma.

Epithelial-derived growth factors such as transforming growth factor-β1 (TGF-β1) and platelet-derived growth factor further influence keratocyte survival, fibroblast differentiation, and stromal remodeling [36]. Through these signals, the epithelium serves as an immunological interface that governs macrophage recruitment, activation state, and downstream fibrotic potential during corneal wound healing.

#### 2.1.2. Bowman’s Layer and Corneal Stroma

Bowman’s layer forms an acellular interface between the epithelium and stroma and contributes to epithelial–stromal signaling during injury. Beneath this layer, the corneal stroma accounts for the majority of corneal thickness and is the principal determinant of biomechanical strength and optical transparency [1,2]. Stromal transparency relies on the precise organization of collagen fibrils and proteoglycans, which minimize light scattering while maintaining tissue integrity [3].

Resident stromal keratocytes maintain ECM homeostasis under physiological conditions. Following injury, keratocytes undergo apoptosis or activation and can differentiate into fibroblasts and contractile myofibroblasts under the influence of cytokines such as TGF-β [7]. This transition represents a key decision point between regenerative repair and fibrotic scarring. Macrophages play a central regulatory role in this process by controlling fibroblast activation, myofibroblast persistence, and ECM remodeling through temporally regulated cytokine and protease release [27]. Persistent macrophage-driven signaling sustains myofibroblast survival and disorganized matrix deposition, thereby promoting pathological fibrosis. Because macrophages interact extensively with stromal cells and the ECM, alterations in stromal architecture directly influence macrophage persistence, polarization, and fibrotic signaling during wound healing.

A pre-Descemet stromal zone, often referred to as Dua’s layer, is a distinct collagen-rich region between the posterior stroma and Descemet’s membrane. While this structure may have biomechanical relevance, its classification as a discrete anatomical layer remains controversial, with many studies considering it a specialized portion of the posterior stroma rather than a separate entity [37].

#### 2.1.3. Descemet’s Membrane and Corneal Endothelium

Descemet’s membrane and the corneal endothelium form the posterior corneal interface and are essential for maintaining stromal dehydration and transparency [4]. Although these layers are not primary sites of immune infiltration, endothelial–stromal interactions contribute indirectly to stromal homeostasis and may influence inflammatory resolution through biomechanical and paracrine signaling [38,39,40]. Endothelial dysfunction can lead to stromal edema and altered matrix mechanics, indirectly affecting immune cell behavior during chronic injury [41].

### 2.2. Corneal Avascularity and Angiogenic Balance

A defining feature of the healthy cornea is its avascularity, which is essential for optical clarity and immune privilege. This state is actively maintained through a balance between pro-angiogenic and anti-angiogenic signals, including vascular endothelial growth factor (VEGF) and basic fibroblast growth factor (bFGF) [42]. Disruption of this balance by hypoxia, infection, or injury leads to corneal neovascularization [42,43].

Macrophages are central regulators of the angiogenic switch, acting as major sources of vascular endothelial growth factor (VEGF), matrix metalloproteinases (MMPs), and other angiogenic mediators that enable endothelial invasion into the normally avascular stroma [44,45]. During the early phases of wound healing, transient macrophage-driven angiogenic responses may facilitate tissue repair by supporting immune-cell recruitment, matrix remodeling, and restoration of tissue homeostasis. However, failure to resolve these responses results in persistent neovascularization characterized by sustained VEGF signaling, continued inflammatory-cell infiltration, and progressive stromal remodeling. This pathological vascularization disrupts corneal immune privilege, amplifies inflammation, promotes fibroblast activation, and contributes to stromal fibrosis and loss of transparency [46,47]. Although precise therapeutic windows remain incompletely defined, current evidence suggests that anti-angiogenic interventions may be most beneficial once neovascularization becomes persistent rather than during the initial reparative phase of wound healing, when limited vascular responses may contribute to tissue repair [47,48]. Thus, macrophage-mediated control of angiogenesis represents a critical link between immune activation and fibrosis in the cornea.

### 2.3. Immune Cells of the Cornea and Immune Privilege

Although traditionally described as immune privileged, the cornea contains a diverse network of resident immune cells, including macrophages, dendritic cells, mast cells, and lymphocytes. Immune privilege is maintained through avascularity, limited antigen presentation, and active immunoregulatory signaling rather than immune cell exclusion [49].

Macrophages represent the most abundant immune population in the cornea, accounting for approximately half of resident immune cells and localizing primarily to the peripheral stroma and limbus [50]. These cells perform immune surveillance while actively suppressing excessive inflammation to preserve transparency [25].

Importantly, macrophage recruitment represents a key mechanistic link between corneal injury and fibrotic remodeling. Damage to epithelial and stromal cells triggers the release of inflammatory mediators, which promote recruitment of monocyte-derived macrophages from the limbal vasculature into the injured cornea [32,51]. Once recruited, macrophages regulate multiple stages of wound healing through the production of cytokines, growth factors, and matrix-remodeling enzymes. While appropriately timed macrophage responses support debris clearance, resolution of inflammation, and tissue repair, persistent macrophage activation sustains TGF-β signaling, fibroblast-to-myofibroblast differentiation, extracellular matrix deposition, and stromal stiffening [52,53]. Consequently, the magnitude, phenotype, and duration of macrophage recruitment are major determinants of whether corneal wound healing progresses toward regenerative repair or pathological fibrosis [52].

### 2.4. Macrophages in Corneal Homeostasis and Injury

In the uninjured cornea, resident macrophages, many of embryonic origin, play a key role in maintaining immune quiescence, clearing apoptotic debris, and supporting long-term tissue integrity [25,54]. This immunoregulatory function is particularly important in corneal transplantation, where macrophage-mediated immune control contributes to graft survival [55].

Following injury, infiltrating monocyte-derived macrophages collaborate with resident populations to initiate inflammation, activate stromal keratocytes, and coordinate repair [7,11]. Early macrophage responses support pathogen clearance and matrix turnover; however, prolonged or improperly resolved macrophage activation sustains fibroblast-to-myofibroblast differentiation, excessive ECM deposition, and increased stromal stiffness, driving irreversible fibrosis [7,27,33].

Macrophages have traditionally been described as either pro-inflammatory (M1) or anti-inflammatory/pro-repair (M2), reflecting their ability to either promote tissue damage or support healing; however, this classification does not capture the full spectrum of macrophage states present in the cornea. Recent single-cell transcriptomic studies have revealed substantial immune-cell heterogeneity within the cornea, including multiple resident macrophage populations with distinct transcriptional signatures and putative functional roles [56,57]. For example, Yaman et al. identified three resident macrophage subsets distinguished by differential expression of Timd4, Lyve1, Folr2, and Ccr2, suggesting differences in tissue residency, phagocytic activity, immune regulation, and inflammatory responsiveness. These findings highlight the limitations of the traditional M1/M2 framework and support the concept that corneal macrophages exist along a dynamic continuum of functional states rather than discrete polarization categories [57]. Earlier studies identifying functionally distinct CCR2^−^ and CCR2^+^ macrophage populations further support this concept of macrophage heterogeneity in the cornea (Table 1) [51,58].

## 3. Corneal Diseases and Wound Healing Responses

Corneal diseases comprise a diverse group of pathological conditions, including traumatic injury, infectious keratitis, and chronic inflammation, all of which disrupt corneal homeostasis and threaten optical clarity. Regardless of the underlying cause, corneal injury initiates a highly coordinated wound-healing response that integrates epithelial repair, stromal remodeling, immune cell recruitment, and ECM reorganization. When tightly regulated, this process restores transparency and function; however, dysregulation at any stage can result in persistent inflammation, fibrotic remodeling, and irreversible visual impairment [7,11].

### 3.1. Epithelial Injury and Regeneration

Injury to the corneal epithelium initiates a rapid and spatially controlled inflammatory response that serves as the first step in tissue repair. This response involves activation of limbal stem cells (LSCs), which proliferate and migrate to resurface the wounded cornea [11,53]. Experimental studies using rabbit cornea models have demonstrated that epithelial migration proceeds at a rate of approximately 100 μm per hour, enabling relatively rapid closure of epithelial defects under physiological conditions [11].

Epithelial restoration occurs in stages, beginning with flattened epithelial cells that provide temporary barrier function. Full epithelial maturation, including cellular differentiation and re-establishment of specialized adhesion complexes, occurs over subsequent weeks and lags initial wound closure. Incomplete restoration of epithelial differentiation or adhesion predisposes the cornea to recurrent erosions and chronic inflammation [7].

### 3.2. Early Cytokine Signaling and Stromal Activation

Epithelial and endothelial injury rapidly induces the release of interleukin-1α (IL-1α) and interleukin-1β (IL-1β), cytokines that are constitutively expressed by corneal cells and released upon cellular damage. These mediators diffuse into the stroma and engage IL-1 receptors on keratocytes, initiating inflammatory signaling cascades that recruit immune cells and prime the tissue for remodeling [52,59]. IL-1 signaling serves as a critical molecular link between epithelial damage and stromal response by regulating keratocyte survival, activation, and interactions with infiltrating immune cells [11].

### 3.3. Keratocyte Apoptosis and Remodeling Initiation

One of the earliest stromal responses to corneal injury is the selective apoptosis of keratocytes adjacent to the damaged region. This process is regulated by local IL-1 concentrations and mediated in part through the Fas–Fas ligand pathway, which is constitutively present in corneal tissue [60].

Controlled keratocyte loss facilitates removal of damaged cells and enables stromal reorganization; however, excessive or prolonged apoptosis destabilizes stromal architecture and amplifies inflammatory signaling. The balance between keratocyte death, repopulation, and differentiation into fibroblasts or myofibroblasts therefore represents a critical decision point between regeneration and fibrosis [7].

### 3.4. From Regeneration to Fibrosis

Under normal conditions, epithelial repair and stromal remodeling are tightly synchronized to restore transparency. In contrast, persistent epithelial defects, sustained cytokine release, or prolonged immune activation can redirect healing toward fibrosis. This pathological state is characterized by myofibroblast persistence, excessive ECM deposition, and increased stromal stiffness, leading to irreversible loss of transparency [11,53].

Emerging evidence identifies macrophages as key regulators of this transition, integrating epithelial-derived signals and stromal cues to shape healing outcomes. Importantly, the timing of macrophage activation and resolution has emerged as a decisive factor: early, transient responses support repair, whereas delayed or sustained macrophage activity promotes chronic inflammation and fibrotic scarring [11]. This temporal framework provides a foundation for therapeutic strategies aimed at preventing corneal fibrosis by modulating immune dynamics rather than suppressing inflammation indiscriminately.

## 4. Corneal Fibrosis: Mechanisms and Pathological Remodeling

Corneal fibrosis is a pathological outcome of wound healing in which repair processes become dysregulated, leading to excessive ECM deposition, disruption of stromal architecture, and permanent loss of corneal transparency. Although fibrotic responses may initially provide structural stabilization following severe injury, their persistence interferes with normal tissue organization and results in lasting visual impairment. Fibrosis is therefore a principal pathological driver of corneal opacity and a major contributor to irreversible corneal blindness [7,11].

Distinguishing regenerative repair from fibrotic remodeling depends on tightly coordinated interactions among epithelial cells, stromal keratocytes, immune cells, and ECM components. Failures in immune resolution, prolonged activation of stromal fibroblasts, and sustained profibrotic signaling, particularly involving TGF-β, shift the healing response toward scarring rather than restoration of transparency [7,27]. Elucidating the molecular and cellular mechanisms that regulate this transition is therefore essential for developing therapies capable of preventing fibrosis while preserving necessary repair functions.

### 4.1. Definition and Hallmarks of Corneal Fibrosis

Corneal fibrosis is defined by the pathological replacement of the native, highly organized stromal architecture with a disordered ECM enriched in fibrillar collagens, fibronectin, and proteoglycans. This aberrant matrix disrupts the precise collagen fibril spacing and uniform diameter that are required for corneal transparency, resulting in increased light scattering and loss of optical clarity [1,7].

At the cellular and molecular levels, corneal fibrosis is characterized by several defining features, including the persistence of activated stromal fibroblasts and myofibroblasts, excessive and prolonged deposition of ECM components, increased stromal stiffness, and failure of fibroblasts to revert to a quiescent keratocyte phenotype following injury [11]. In more severe or chronic disease states, fibrotic remodeling is often accompanied by sustained inflammation and pathological neovascularization, further exacerbating tissue disorganization and visual impairment [7,46].

Clinically, these pathological changes manifest as stromal haze, scarring, irregular astigmatism, and reduced visual acuity, which may persist long after epithelial closure has been achieved. Importantly, the temporal dissociation between epithelial repair and stromal remodeling underscores that corneal fibrosis represents a distinct disease process rather than a simple delay in wound healing [53]. While these hallmarks are consistently observed across experimental models and clinical specimens, the relative contribution of individual pathways varies depending on injury type, severity, and disease context.

### 4.2. Cellular Drivers of Corneal Fibrosis

Fibrotic remodeling of the cornea is driven primarily by maladaptive activation of stromal cells following injury. In the uninjured cornea, keratocytes exist in a quiescent state and are responsible for maintaining ECM homeostasis. After injury, keratocytes near the wound edge undergo apoptosis, while surviving cells become activated in response to epithelial- and immune-derived cytokines, most notably IL-1 and TGF-β [7,11].

Activated keratocytes differentiate into stromal fibroblasts that migrate into the injured region and deposit ECM to stabilize the tissue. During regenerative healing, these fibroblasts are transient and either undergo apoptosis or revert to a keratocyte phenotype during the remodeling phase. In contrast, fibrotic healing is characterized by further differentiation of fibroblasts into α-smooth muscle actin (α-SMA)-expressing myofibroblasts, which display enhanced contractility and sustained matrix synthesis [7,8].

Persistent myofibroblasts are the principal cellular mediators of corneal fibrosis. Their continued presence is closely associated with stromal opacity and increased tissue stiffness, as they secrete disorganized collagen and fibronectin while exerting mechanical tension on the surrounding matrix. Failure to eliminate myofibroblasts during the resolution phase is therefore a defining feature of pathological corneal scarring [11,27].

### 4.3. Molecular and Mechanical Drivers of Fibrotic Remodeling

The shift from regenerative repair to fibrosis is regulated by sustained activation of profibrotic signaling networks rather than a single molecular trigger. Among these pathways, TGF-β signaling plays a central role by promoting myofibroblast differentiation, α-SMA expression, collagen production, and resistance to apoptosis [7,61]. However, the effects of TGF-β signaling are highly context- and timing-dependent, and transient activation is also required for normal stromal repair, underscoring the importance of regulated resolution rather than complete inhibition.

Inflammatory cytokines released early after injury, including IL-1, TNF-α, and IL-6, indirectly contribute to fibrosis by amplifying immune cell recruitment and prolonging stromal activation when resolution fails to occur [11]. MMPs are essential mediators of corneal wound healing because they degrade damaged ECM, facilitate epithelial and stromal cell migration, and enable matrix remodeling; however, excessive or prolonged MMP activity can destabilize stromal architecture and contribute to pathological remodeling [62].

Mechanical cues further reinforce fibrotic progression. Early deposition of abnormal ECM increases stromal stiffness, which in turn promotes myofibroblast differentiation through mechanotransduction pathways involving integrins and cytoskeletal tension. This creates a positive feedback loop in which biochemical and biomechanical signals mutually reinforce fibrosis [27].

### 4.4. Temporal Dysregulation of Wound Healing

Importantly, corneal fibrosis is not an inevitable consequence of injury but arises from disrupted timing within the wound-healing cascade. Acute inflammation and stromal activation are necessary for repair; however, failure to resolve these responses shifts healing toward pathological remodeling [11].

During normal healing, inflammatory signaling diminishes as epithelial integrity is restored, allowing fibroblasts and immune cells to exit the wound environment and enabling re-establishment of keratocyte quiescence. In fibrotic healing, inflammatory and profibrotic cues persist beyond the proliferative phase, sustaining fibroblast activation and preventing normal stromal resolution [7].

This temporal mismatch, where signals appropriate for early repair remain active during later remodeling, has emerged as a unifying concept in fibrotic disease. Importantly, macrophage timing and stromal stiffness appear to contribute to distinct stages of fibrosis progression. During fibrosis initiation, excessive or prolonged inflammatory macrophage activation increases IL-1β, TNF-α, TGF-β, and MMP activity, thereby promoting keratocyte activation, fibroblast recruitment, and myofibroblast differentiation. During fibrosis maintenance, abnormal ECM deposition progressively increases stromal stiffness, which reinforces integrin-mediated mechanotransduction and supports continued myofibroblast survival. Increased matrix stiffness may also perpetuate macrophage retention and profibrotic activation, creating a feed-forward loop that sustains stromal haze and fibrosis even after the initial injury response has diminished. Increasing evidence suggests that immune cells, particularly macrophages, serve as critical temporal regulators of this transition by integrating epithelial, stromal, and inflammatory inputs to determine whether healing resolves or progresses toward scarring [27,33].

Macrophage functions represent dynamic transcriptional and functional programs rather than fixed phenotypes. Transitions between phases are governed by temporal cues and microenvironmental signals rather than discrete macrophage subtypes (Table 2) [7,63,64].

These insights are derived largely from preclinical corneal injury models in which macrophage recruitment, depletion, or polarization is experimentally manipulated; confirmation of analogous temporal roles in human corneal fibrosis is still emerging.

### 4.5. Clinical Relevance and Therapeutic Limitations

Corneal fibrosis remains a major therapeutic challenge in the management of corneal injury and disease. Current pharmacologic treatments, including corticosteroids and broad-spectrum anti-inflammatory agents, primarily suppress inflammation but do not adequately address the cellular and molecular mechanisms that sustain fibrotic remodeling. Consequently, these approaches often fail to reverse established stromal scarring or restore normal ECM [7,11]. Surgical interventions such as phototherapeutic keratectomy or corneal transplantation can improve vision in advanced cases but are invasive, associated with significant risks, and do not prevent recurrent fibrosis in inflammatory or immune-mediated conditions.

Increasing evidence suggests that these limitations arise in part from an incomplete ability to regulate macrophage-driven immune dynamics during corneal wound healing. Macrophages function as central coordinators of inflammation, stromal remodeling, and resolution by integrating epithelial-derived signals, ECM cues, and cytokine networks [25,27]. Importantly, non-selective immunosuppression may impair beneficial macrophage functions, such as apoptotic cell clearance, matrix remodeling, and resolution signaling, thereby hindering regenerative repair while failing to prevent fibrotic progression.

In this context, regenerative strategies that modulate macrophage behavior through paracrine and EV-mediated mechanisms have gained increasing attention. MSC-derived EVs have been shown to carry bioactive proteins involved in immune regulation, matrix remodeling, and wound resolution, including factors capable of influencing macrophage activation states. To date, these effects have been demonstrated predominantly in preclinical corneal injury models, and robust clinical evidence supporting EV-mediated macrophage modulation in human corneal fibrosis remains limited.

Recent proteomic analyses of EVs in corneal regenerative medicine have identified enrichment of anti-inflammatory, anti-fibrotic, and immunoregulatory proteins, highlighting EVs as biologically active mediators rather than passive delivery vehicles [18]. By shaping macrophage responses toward pro-resolution and anti-fibrotic programs, EV-based therapies offer a cell-free approach to restore appropriate immune timing while avoiding many of the risks associated with live cell transplantation.

Collectively, these findings support a conceptual shift in corneal fibrosis therapy, from indiscriminate suppression of inflammation toward precise temporal regulation of macrophage function, potentially achieved through MSC- and EV-based interventions. Such approaches position macrophages not solely as drivers of pathology but as tractable therapeutic targets capable of promoting regeneration when appropriately instructed.

## 5. Macrophages as Central Regulators of Corneal Healing and Fibrosis

Macrophages are phagocytes of the innate immune system with core functions in debris clearance, antimicrobial defense, and coordination of tissue repair. Rather than behaving as simple effector cells, corneal macrophages act as integrators of epithelial danger signals, stromal remodeling cues, and vascular/immune-privilege checkpoints, thereby shaping whether healing returns toward transparency or deviates toward persistent haze and fibrosis [11,25,49].

A central theme emerging from experimental injury models and high-dimensional profiling is that macrophage impact is governed less by the mere presence or absence of macrophages and more by when particular macrophage programs dominate. Early inflammatory functions can be indispensable for repair, while delayed resolution or prolonged activation can sustain myofibroblasts, amplify profibrotic cytokine loops, and maintain a matrix environment that is incompatible with stromal reorganization [7,27,33].

### 5.1. Origin and Heterogeneity of Corneal Macrophages

The cornea is often described as immune-privileged tissue not due to an absence of immune cells, but rather the presence of a unique local environment and specialized myeloid cells that actively promote immune tolerance and limit inflammation to preserve tissue clarity and vision [25]. In the normal murine corneal stroma, many resident leukocytes are of monocyte/macrophage lineage, and roughly half of CD45^+^ stromal immune cells express the macrophage-associated marker F4/80, underscoring macrophages as a dominant resident immune population in steady state [50]. Human studies also support a well-established resident immune landscape, and modern in vivo imaging and systems-level profiling continue to revise older assumptions that the central cornea is “immune empty” [65].

Corneal macrophages arise from distinct developmental and recruitment pathways. Tissue-resident macrophages in many organs can originate from embryonic sources (yolk sac/fetal liver) and persist via self-renewal [54]. Resident populations localize mainly to the limbus and peripheral stroma, performing immune surveillance and supporting epithelial integrity; in parallel, circulating monocytes can infiltrate tissues and differentiate into macrophages within the corneal stroma in response to inflammatory signals through chemokines such as CCL2/MCP-1, CXCL8, and VEGF [66]. These recruited macrophages coexist with resident populations but often exhibit distinct transcriptional profiles, activation thresholds, and functional roles. The relative contribution of resident versus recruited macrophages varies depending on injury severity, duration, and microenvironmental cues [25,49].

Importantly, accumulating evidence indicates that resident macrophages and monocyte-derived macrophages play non-redundant and temporally distinct roles in corneal wound healing and fibrosis. Tissue-resident macrophages, which arise from embryonic progenitors and persist through self-renewal, are thought to function primarily as immune surveillants and regulators of tissue homeostasis, contributing to immune privilege, debris clearance, and early containment of inflammation in the steady-state cornea [25,54]. These resident populations appear particularly important for maintaining stromal quiescence and facilitating timely resolution following mild or self-limited injury.

In contrast, monocyte-derived macrophages are rapidly recruited from the circulation in response to epithelial damage, chemokine gradients, and vascular leakage at the limbus. These cells dominate during acute and severe injury and are major sources of inflammatory cytokines, growth factors, and matrix-modifying enzymes that drive early defense and stromal remodeling [25,49]. While transient recruitment of monocyte-derived macrophages is essential for effective repair, their persistence or delayed resolution has been strongly associated with sustained myofibroblast activation, excessive ECM deposition, angiogenesis, and fibrotic scarring [27,33].

Taken together, these observations indicate that corneal fibrosis is not simply a consequence of macrophage presence, but rather reflects a failure to properly transition from recruited inflammatory macrophage programs toward resident-like or resolution-phase states. This distinction reinforces the concept that therapeutic strategies should aim to restore appropriate macrophage balance and timing, limiting prolonged monocyte-derived inflammatory activity while preserving or enhancing the regulatory functions of resident macrophages, rather than globally suppressing macrophage populations.

Macrophage activation is often described using the classical (M1) versus alternative (M2) framework, a simplified model that has been widely used in immunology to categorize stimulus-dependent macrophage programs based on surface markers, cytokine/chemokine output, and effector functions [67]. Although useful as a conceptual starting point, this paradigm is increasingly recognized as an oversimplification of a broader continuum of macrophage states shaped by tissue context and timing [68,69].

M1-like macrophages are typically associated with early inflammatory defense. They are induced by signals such as interferon-γ (IFN-γ), lipopolysaccharide (LPS), and TNF, and they promote antimicrobial activity and Th1-skewing immunity through production of pro-inflammatory cytokines, chemokines, and reactive nitrogen intermediates (e.g., nitric oxide). These responses are critical for host defense, but when excessive or prolonged they can amplify tissue inflammation and damage [70].

In contrast, M2 macrophages are broadly associated with inflammation resolution, immune regulation, and tissue repair; however, they do not represent a single homogeneous population. Instead, M2 macrophages are commonly subdivided into M2a, M2b, M2c, and M2d subtypes based on distinct inducing signals and functional properties [27,58]. M2a macrophages are induced primarily by interleukin-4 (IL-4) or interleukin-13 (IL-13) and are involved in wound healing, Th2-associated immune responses, and ECM remodeling. These cells secrete mediators such as IL-10, TGF-β, CCL17, and CCL22 and can support tissue repair; however, prolonged M2a activity has also been implicated in fibrotic remodeling due to sustained matrix production. M2b macrophages arise in response to immune complexes in combination with Toll-like receptor ligands and IL-1β and exhibit a mixed regulatory phenotype, producing both anti-inflammatory cytokines such as IL-10 and pro-inflammatory mediators including TNF-α, IL-1β, IL-6, and CCL1. Through this balanced cytokine output, M2b macrophages regulate the magnitude and breadth of immune responses rather than fully suppressing inflammation. M2c macrophages, induced by IL-10, TGF-β, and glucocorticoids, are associated with immune deactivation, tissue remodeling, and efficient clearance of apoptotic cells through efferocytosis, functions that are critical for terminating inflammation and restoring tissue homeostasis [71,72]. Finally, M2d macrophages have been described in angiogenic microenvironments and are associated with vascular growth and tissue remodeling programs, linking this subtype to pathological angiogenesis when regulatory constraints fail (Table 3) [73,74,75,76].

Although the M1/M2 states remain useful for conceptualizing macrophage plasticity, they do not fully capture the diversity of macrophage states observed in vivo, particularly within specialized tissues such as the cornea. Increasing evidence indicates that macrophages occupy a dynamic continuum of activation states shaped by local cytokine milieus, metabolic conditions, neural inputs, and temporal context during wound healing [25,68,69].

Advances in single-cell transcriptomic technologies have transformed the study of complex tissues by enabling high-resolution characterization of cellular heterogeneity under both physiological and pathological conditions. In the context of the ocular surface, these approaches are particularly valuable given the cornea’s immune-privileged status, low cellularity, and dynamic responses to injury and disease. Single-cell RNA sequencing allows unbiased identification of immune and stromal cell populations, detection of rare or transient cell states, and mapping of injury- or disease-associated transcriptional programs that are not discernible using bulk transcriptomic methods. Importantly, recent studies highlight the potential of single-cell approaches to identify disease-associated immune signatures and candidate biomarkers relevant to diagnosis, prognosis, and therapeutic targeting in ocular surface disorders. By resolving macrophage diversity, activation states, and temporal dynamics during homeostasis and pathology, single-cell transcriptomics provides a powerful framework for understanding how specific macrophage programs contribute to regeneration versus fibrosis in the cornea [56].

Quantitative analyses of corneal macrophage subsets have provided direct evidence that distinct macrophage populations play temporally specialized roles during epithelial wound healing. Using flow cytometry-sorted macrophages followed by quantitative polymerase chain reaction (qPCR), Liu and colleagues demonstrated that CD64^+^CCR2^+^ corneal macrophages preferentially express pro-inflammatory genes characteristic of inflammatory macrophage programs, including Il1b and Tnf. In contrast, CD64^+^CCR2^−^ macrophages exhibit elevated expression of genes associated with inflammation suppression and tissue repair, such as Il10, Arg1, Mrc1, Mgl1, Mgl2, Ym1, and Fizz1 [51]. Although several of these markers were originally characterized in macrophages from other tissues, their expression in the cornea reflects conserved functional programs related to immune regulation, efferocytosis, and tissue repair rather than rigid tissue-specific identities. In the corneal microenvironment, these markers therefore serve as functional correlates of pro-resolving macrophage activity rather than definitive polarization labels. Functional depletion experiments further revealed that removal of CD64^+^CCR2^+^ macrophages reduced neutrophil recruitment and inflammatory cytokine expression following corneal epithelial injury, whereas depletion of CD64^+^CCR2^−^ macrophages led to exaggerated neutrophil influx and heightened inflammatory signaling. Notably, depletion of either subset resulted in delayed epithelial wound closure, indicating that both inflammatory and regulatory macrophage populations are required for balanced and effective corneal repair, with distinct roles at different stages of healing [51].

Subsequent work extended these findings by uncovering a previously unappreciated layer of neuro-immune regulation governing macrophage function in the cornea. Distinct corneal macrophage subsets were found to differentially express autonomic nervous system receptors, with CD64^+^CCR2^−^ macrophages preferentially expressing the α7 nicotinic acetylcholine receptor (α7nAChR) and CD64^+^CCR2^+^ macrophages enriched for the β2-adrenergic receptor (β2AR) [58]. Pharmacologic modulation of these pathways demonstrated functional consequences: topical administration of a β2AR agonist enhanced pro-inflammatory gene expression in CD64^+^CCR2^+^ macrophages isolated from injured corneas, whereas stimulation of α7nAChR selectively increased anti-inflammatory gene expression in CD64^+^CCR2^−^ macrophages. These findings establish that local autonomic signaling actively tunes macrophage inflammatory versus regulatory programs during corneal repair, reinforcing the concept that macrophage behavior is shaped by integrated epithelial, immune, and neural cues rather than by intrinsic lineage alone [58].

Single-cell transcriptomic analyses reveal multiple macrophage populations defined by distinct transcriptional programs related to antigen presentation, cytokine secretion, ECM turnover, angiogenic regulation, and immune resolution (Table 4) [25,49,56,57,65,77]. These findings emphasize that macrophage heterogeneity in the cornea is best understood as a context- and time-dependent continuum, rather than as fixed activation states. Macrophage activation states are dynamically shaped by local cytokine environments, metabolic constraints, neural inputs, and the evolving tissue microenvironment during wound healing, and inappropriate persistence of inflammatory programs can disrupt immune balance and promote fibrosis and loss of transparency [25,49].

### 5.2. Early Macrophage Responses Are Essential for Corneal Repair

Immediately following corneal injury, macrophages act as first responders that initiate and coordinate early wound-healing events. Across experimental models of epithelial abrasion, alkali burn, infectious keratitis, and surgical injury, monocyte-derived macrophages rapidly infiltrate the corneal stroma within hours to days after damage. Together with neutrophils, these early macrophages clear pathogens, phagocytose apoptotic keratocytes, and remove cellular debris [7,11].

Loss-of-function studies have provided direct evidence for the necessity of this early macrophage response. Pharmacologic depletion or genetic ablation of macrophages results in delayed epithelial closure, impaired stromal remodeling, defective ECM organization, and increased susceptibility to infection, underscoring that macrophage activity during the acute inflammatory phase is required for effective repair rather than being inherently pathological [11,25].

Beyond debris clearance, early macrophages function as signaling control that orchestrate epithelial–stromal crosstalk. By releasing cytokines such as interleukin-1β, tumor necrosis factor-α, and interleukin-6, macrophages amplify epithelial-derived danger signals and activate stromal keratocytes. These signals promote fibroblast migration and deposition of ECM, stabilizing the wounded tissue. Concurrently, macrophage-derived matrix metalloproteinases degrade damaged matrix components, facilitating cell migration and preventing accumulation of dysfunctional ECM [11,61]. When confined to this early temporal window, inflammatory and matrix-remodeling functions support regeneration rather than fibrosis.

Single-cell transcriptomic analyses reinforce this concept by demonstrating that early corneal macrophages are transcriptionally programmed for antimicrobial defense, cytokine signaling, and matrix turnover rather than fibrotic activity. These early-response populations express innate immune and wound-surveillance gene programs optimized for rapid tissue defense, highlighting that macrophage-driven inflammation is a prerequisite for successful corneal healing when appropriately constrained in time [49].

### 5.3. Resolution-Phase Macrophages and Anti-Fibrotic Functions

As corneal wound healing progresses, macrophages undergo a critical functional transition from inflammatory activation toward resolution and tissue restoration. Resolution-phase macrophages actively suppress ongoing inflammation, promote epithelial differentiation, and limit stromal fibrosis. This transition is driven by changes in the cytokine milieu, uptake of apoptotic cells, and restoration of epithelial integrity, resulting in downregulation of pro-inflammatory mediators and increased production of anti-inflammatory and pro-resolving factors [27].

A defining anti-fibrotic role of resolution-phase macrophages is their regulation of myofibroblast fate. During regenerative healing, macrophages promote apoptosis or de-differentiation of α-smooth muscle actin-positive myofibroblasts, enabling re-establishment of a quiescent keratocyte population. Failure of this macrophage-mediated clearance has been directly associated with persistent stromal haze and fibrosis in multiple corneal injury models. In parallel, macrophages contribute to ECM remodeling by facilitating collagen reorganization and clearance of excess matrix, processes essential for recovery of stromal transparency [27,61].

Importantly, resolution-phase macrophages represent a distinct functional program rather than simply a suppressed version of early inflammatory macrophages. Transcriptomic studies reveal enrichment of pathways related to phagocytosis, lipid metabolism, efferocytosis, and tissue repair rather than cytokine amplification. This reparative macrophage state emerges only when inflammatory cues subside, emphasizing that prevention of fibrosis depends on a timely macrophage transition, not on global suppression of immune activity [25,27].

### 5.4. When Macrophages Drive Corneal Fibrosis

Macrophages become pathogenic drivers of corneal fibrosis when inflammatory activation persists beyond the appropriate temporal window. In severe chemical injury, chronic infection, autoimmune disease, or repeated surgical trauma, macrophages maintain a pro-inflammatory and pro-fibrotic secretory profile well into the proliferative and remodeling phases. Under these conditions, macrophage-derived cytokines and growth factors reinforce fibroblast-to-myofibroblast differentiation, sustain ECM overproduction, and prevent resolution [7,11].

Mechanistically, inflammasome activation within infiltrating macrophages has emerged as a key driver of fibrotic signaling. Activation of the NLRP3 inflammasome enhances IL-1β release, which in turn upregulates epithelial TGF-β1 expression, creating a feed-forward loop that sustains myofibroblast survival and collagen deposition. Genetic or pharmacologic inhibition of NLRP3 signaling reduces stromal haze and attenuates fibrosis in murine models, confirming its pathogenic role [33].

Macrophages also indirectly promote fibrosis by driving angiogenesis and lymphangiogenesis, processes that disrupt corneal immune privilege and perpetuate inflammatory cell influx. These vascular changes further destabilize the corneal microenvironment, shifting healing from regenerative to pathological remodeling [44,46].

In this context, macrophage migration inhibitory factor (MIF) has been identified as an upstream regulator of sustained macrophage activation in ocular tissues. MIF promotes macrophage retention and amplifies pro-inflammatory signaling through NF-κB and STAT3 pathways, reinforcing IL-1β and TNF-α production while actively opposing macrophage deactivation and resolution programs. These findings position MIF as a molecular checkpoint that locks macrophages into a pathogenic state and highlight it as a promising therapeutic target for restoring immune timing and limiting fibrosis [78].

### 5.5. Therapeutic Implications of Macrophage Timing

Recognition of macrophages as temporal regulators of corneal healing has profound therapeutic implications. Broad immunosuppression or macrophage depletion may be detrimental if applied during the early inflammatory phase, as it can impair epithelial closure and stromal repair. In contrast, interventions that promote macrophage resolution or reprogramming during later stages offer substantial promise for preventing or reversing fibrosis [27].

Experimental corneal injury models indicate that macrophage immunomodulation must be temporally aligned with distinct phases of wound healing. Pro-inflammatory macrophage programs predominate during the initial hours to early days following injury, where they are essential for pathogen clearance, apoptotic cell removal, and initiation of epithelial–stromal signaling. As epithelial integrity is progressively restored, macrophages undergo a functional transition toward resolution-phase programs that suppress inflammation, promote myofibroblast clearance, and facilitate ECM reorganization. Fibrosis emerges when this transition is delayed or incomplete, resulting in prolonged inflammatory or profibrotic macrophage signaling. Importantly, these transitions are governed by dynamic cytokine and microenvironmental cues rather than rigid temporal thresholds, supporting therapeutic strategies that modulate macrophage signaling and timing rather than enforcing static phenotypic identities.

Regenerative strategies increasingly focus on modulating macrophage timing rather than indiscriminately suppressing inflammation. MSC-based therapies and their EVs have been shown to re-educate macrophages toward anti-inflammatory, pro-resolving states in corneal injury models. These approaches reduce myofibroblast persistence, normalize ECM organization, and preserve transparency without compromising early immune defense [26,29,79].

Ultimately, timing-based macrophage modulation represents a conceptual shift in corneal fibrosis therapy. By aligning therapeutic intervention with specific phases of wound healing, macrophages can be harnessed as agents of regeneration rather than drivers of scarring. Although this review emphasizes macrophage timing as a central determinant of regenerative versus fibrotic outcomes, corneal fibrosis does not arise from immune dysregulation in isolation. Increasing evidence indicates that biomechanical changes within the corneal stroma, particularly progressive ECM stiffening driven by persistent myofibroblast activity, can reinforce and prolong profibrotic macrophage programs. In this context, macrophage timing acts as an upstream regulatory switch that initiates either resolution or fibrosis, while stromal mechanical cues stabilize and amplify macrophage persistence once fibrotic remodeling is established. This bidirectional immune–mechanical feedback underscores that effective anti-fibrotic strategies must restore both immune resolution and stromal normalization, rather than targeting either process independently. Together, these observations reframe corneal fibrosis not as an inevitable consequence of injury, but as a preventable failure of immune resolution. Therapeutic strategies targeting macrophages are to be most effective when applied in a phase-specific manner, supporting essential early inflammatory functions while accelerating the timely transition toward resolution and preventing late-stage profibrotic persistence. Macrophage functional programs evolve dynamically across the wound-healing continuum, from early inflammation to resolution or fibrotic progression. This temporal framework is supported by foundational work defining phase-specific macrophage roles in tissue repair and fibrosis, which demonstrates that dysregulation of macrophage timing, rather than macrophage activation *per se*, is a key driver of pathological scarring [27].

## 6. Mesenchymal Stromal Cells as Modulators of Macrophage Timing

### 6.1. Immunomodulatory Properties of MSCs

MSCs are multipotent, fibroblast-like progenitor cells first described in the bone marrow and later identified in many adult and perinatal tissues, including adipose tissue and umbilical cord [80]. To integrate how these cells are defined across studies, the International Society for Cellular Therapy proposed minimal criteria, plastic adherence in standard culture, characteristic surface marker expression (commonly including CD73, CD90, and CD105), and tri-lineage differentiation potential, now widely used to standardize MSC identity across tissues and laboratories [81].

Beyond differentiation, MSCs are now primarily viewed as “environmental regulators” that reshape tissue injury niches through secreted mediators and contact-dependent immune interactions. Across multiple systems, MSCs suppress excessive adaptive immune activation by limiting effector T-cell responses, altering B-cell activity, and promoting regulatory T-cell programs [82,83]. At the same time, MSCs modulate innate immunity by reprogramming monocytes/macrophages and influencing dendritic cell and NK cell function, shifting inflammatory circuits toward resolution [83,84]. This is highly relevant to corneal repair, where immune responses must be sufficiently strong to protect the tissue yet sufficiently constrained to preserve transparency. In a murine alkali-burn model, subconjunctival MSC delivery reduced opacity and inflammatory infiltration while altering macrophage-associated inflammatory readouts in the injured cornea [79].

MSC signaling can also indirectly shape macrophage dynamics by modifying stromal cell behavior. Once keratocytes are activated, they produce cytokines and matrix signals that sustain leukocyte recruitment and can perpetuate fibrosis. In this context, MSC-conditioned media has been shown to alter keratocyte activation-associated behaviors, supporting the idea that MSC paracrine factors can reduce the stromal “feed-forward” signals that maintain macrophage influx and inflammatory persistence. Together, these findings position MSCs as phase-sensitive immunomodulators capable of influencing both the initial and subsequent resolution kinetics of corneal inflammation [79,85].

### 6.2. MSC–Macrophage Crosstalk in Corneal Repair

Research now supports the concept that one of the most therapeutically relevant MSC outputs is not simply “immunosuppression,” but targeted reprogramming of macrophage fate and function. A key mechanistic axis involves MSC-derived interleukin-1 receptor antagonist (IL-1Ra), which competitively inhibits IL-1 signaling and can redirect macrophage polarization away from sustained pro-inflammatory programs [86]. IL-1Ra secreted by MSCs has been shown to promote macrophage skewing toward pro-resolving phenotypes and dampen IL-1-driven inflammatory activation, providing a plausible molecular route by which MSCs can accelerate the transition from inflammatory to resolution-phase macrophage states [86].

Eslani and colleagues showed direct evidence for cornea-specific MSC-educated macrophage programs by using cornea-derived MSCs (cMSCs) in models of corneal injury and inflammatory neovascularization. cMSCs shift macrophages toward an immunoregulatory and anti-angiogenic phenotype and reduce macrophage-driven pathological vascular responses that threaten corneal transparency [26]. Mechanistically, they identified pigment epithelial-derived factor (PEDF) as a decisive MSC-derived mediator that constrained macrophage accumulation in the injured cornea by promoting apoptosis of recruited macrophages, thereby preventing prolonged inflammatory residence [26].

In addition to paracrine reprogramming of macrophages, studies of umbilical cord-derived MSCs (UMSCs) have highlighted another important facet of MSC–immune interactions that may shape macrophage behavior during tissue repair. Coulson-Thomas reported that the MSC glycocalyx (a surface matrix rich in glycosaminoglycans) is functionally important for immune modulation and graft persistence; disrupting glycocalyx components reduced the cells’ immunomodulatory effects, supporting a model in which MSC surface structures, alongside soluble factors, shape inflammatory cell recruitment/survival in vivo [87]. In an alkali burn corneal injury model, transplanted human UMSCs suppressed the host inflammatory response and facilitated recovery of corneal transparency within two weeks [87]. M2 polarization of macrophages after MSC administration was also found to be a crucial actor in reducing the risk of rejection after corneal transplantation [88].

## 7. Extracellular Vesicles as Cell-Free Regulators of Fibrosis

Increasing evidence indicates that many of the therapeutic benefits attributed to MSCs are mediated not by direct cell engraftment, but by factors released into the extracellular milieu, collectively referred to as the MSC secretome. This secretome comprises both soluble mediators and membrane-enclosed EVs, including exosomes and microvesicles. Focused characterization of MSC-derived EVs has revealed that these vesicles can transfer complex regulatory information to recipient cells rather than acting as passive byproducts of cellular metabolism [14,17].

Importantly, EVs offer several advantages over live-cell therapies, including improved safety profiles, reduced immunogenicity, easier storage and standardization, and the ability to cross biological barriers [22].

### 7.1. MSC-Derived EVs: Composition and Function in Corneal Repair

Recent studies have begun to define the functional cargo of MSC-derived EVs in the context of corneal repair. EVs isolated from human MSCs derived from either corneal tissue or bone marrow have been shown to promote neurite extension in vitro and significantly enhance corneal nerve regeneration in vivo. Notably, cornea-derived MSC-EVs displayed a distinct microRNA (miRNA) profile enriched for regulators of ECM organization, neurotrophic signaling, and immune modulation, suggesting that EV cargo reflects tissue origin and functional specialization [18].

These findings underscore the concept that MSC-EVs are not interchangeable but can be tailored, either by selecting specific MSC tissue sources or by manipulating culture conditions, to deliver targeted regenerative signals. In the cornea, this is particularly relevant because successful repair requires coordination between nerve regeneration, epithelial restoration, stromal remodeling, and immune resolution. By simultaneously influencing neuronal, stromal, and immune pathways, MSC-EVs represent multifunctional therapeutic agents capable of addressing multiple dimensions of corneal injury.

### 7.2. EV-Mediated Macrophage Reprogramming in the Cornea

Recent research supports the ability of EVs to modulate macrophage activation states, thereby influencing inflammatory resolution and fibrotic outcomes. In one recent study, macrophages conditioned with epidermal growth factor (EGF) produced exosomes that suppressed inflammation. When these exosomes were applied to injured murine corneas, they significantly reduced ocular inflammation and increased expression of arginase-1 (ARG1), a marker associated with anti-inflammatory and pro-resolving macrophage programs, indicating functional macrophage reprogramming in vivo [89].

Although direct investigations of MSC-EV-mediated macrophage polarization specifically in corneal fibrosis remain limited, several corneal studies provide mechanistic insights into MSC-mediated immune regulation. In a diabetic corneal wound-healing model, MSC-derived tumor necrosis factor-stimulated gene-6 (TSG-6) promoted macrophage polarization toward reparative M2 phenotypes and enhanced epithelial wound closure, highlighting a direct link between MSC-secreted factors, macrophage reprogramming, and corneal repair [90]. Similarly, cornea-derived MSCs have been shown to therapeutically modulate macrophage function by suppressing pro-inflammatory and pro-angiogenic macrophage activities, further supporting the role of MSCs in shaping the corneal immune microenvironment [26]. These findings support the concept that MSC-based therapies can influence corneal healing through active regulation of macrophage behavior. While direct evidence linking specific MSC-EV cargo molecules to macrophage polarization in corneal fibrosis remains limited, studies in other injury and fibrosis models have implicated EV-associated miRNAs and immunomodulatory mediators, including miR-146a, miR-223, TSG-6, and PGE2, in promoting pro-resolving macrophage programs and suppressing fibrosis [91,92]. MSC-EVs have been shown to accelerate epithelial wound closure, reduce corneal opacity, and suppress pro-inflammatory cytokine production following injury, consistent with attenuation of innate immune activation [20,21,29]. Additional mechanistic support comes from broader EV translational work and ocular immunology reviews emphasizing EV cargo-driven immune regulation [22,24].

Collectively, these studies position EV therapy as a promising cell-free strategy for controlling macrophage timing and function during corneal wound healing. By limiting prolonged inflammatory macrophage activity and promoting resolution-phase programs, EV-based approaches have the potential to suppress fibrotic remodeling while preserving essential early immune responses. As such, MSC-derived EVs represent a biologically sophisticated and clinically attractive platform for preventing corneal fibrosis through precise immune reprogramming rather than broad immunosuppression.

At a mechanistic level, the ability of EVs to regulate macrophage polarization and timing is largely mediated by their molecular cargo, particularly regulatory proteins and microRNAs (miRNAs). EVs are increasingly recognized as key mediators of intercellular communication through their delivery of bioactive cargos that regulate immune activation, inflammation, and fibrotic remodeling. EV-associated miRNAs can modulate recipient cell gene expression by targeting signaling pathways that govern macrophage polarization, cytokine production, and ECM regulation. Recent reviews have highlighted the central role of EV-miRNA cargo in coordinating inflammatory responses, immune-cell reprogramming, and fibrotic remodeling across multiple tissues, emphasizing their importance as regulators of tissue repair and fibrosis [91]. As comprehensively reviewed by Verma et al. [93], multiple EV-miRNAs have been implicated in the control of inflammatory and fibrotic responses relevant to tissue repair. For example, miR-146a and miR-223 are frequently associated with suppression of excessive Toll-like receptor and NF-κB signaling, thereby limiting prolonged pro-inflammatory macrophage activation and promoting resolution. In contrast, sustained delivery of miR-155 has been linked to reinforcement of pro-inflammatory macrophage programs, underscoring the importance of temporal regulation. EV-associated miRNAs such as members of the miR-29 family have also been shown to inhibit collagen synthesis and profibrotic gene expression in stromal cells, indirectly influencing macrophage–fibroblast crosstalk and attenuating fibrotic progression. Collectively, these findings support a mechanistic model in which EV-miRNA cargo acts as a temporally sensitive regulatory layer capable of reprogramming macrophages and stromal cells toward regenerative or fibrotic outcomes depending on cargo composition and the phase of wound healing during which EV delivery occurs. This framework provides review-level support for the concept that vesicle-mediated communication is a critical determinant of immune resolution and fibrosis during corneal wound healing [93].

### 7.3. Limbal Epithelial Cell-Derived Exosomes and Disrupted Vesicle Signaling in Corneal Fibrosis

Emerging evidence indicates that exosomes derived from limbal epithelial cells (LECs) play a critical role in coordinating intercellular communication within the corneal niche, influencing both stromal cells and immune responses. Human LEC-derived exosomes carry distinct microRNA and protein cargos that regulate limbal stromal cell behavior, including proliferation, stem cell marker expression, and wound-healing responses. Notably, exosomes derived from non-diabetic LECs enhance stromal cell proliferation and wound repair more effectively than those from diabetic donors, suggesting that disease-associated alterations in exosomal cargo contribute to impaired corneal healing in diabetes. Diabetic LEC-derived exosomes also exhibit differences in proteins involved in exosome biogenesis and cargo packaging, which may further compromise epithelial–stromal communication and promote persistent inflammation and dysregulated matrix remodeling conditions that favor fibrotic outcomes [93].

Although direct studies examining macrophage modulation by limbal epithelial exosomes in the cornea remain limited, extensive evidence from other tissues demonstrates that epithelial-derived exosomes can influence macrophage activation, cytokine production, and resolution signaling. Disruption of this epithelial–immune vesicle axis in diabetes may therefore contribute to sustained pro-inflammatory macrophage activity and delayed transition toward resolution-phase programs. Integrating limbal epithelial exosome signaling into a temporal model of corneal wound healing highlights how disease-associated impairments in vesicle-mediated communication can disrupt macrophage timing and promote fibrotic remodeling. Collectively, these findings further support vesicle-based therapeutic strategies aimed at restoring coordinated epithelial–stromal–immune communication as a means to prevent corneal fibrosis, particularly in diabetic corneas.

## 8. Macrophage-Based Therapeutic Strategies for Corneal Fibrosis: Cell Therapy Versus Host Reprogramming

Given the central role of macrophages in determining regenerative versus fibrotic outcomes in the cornea, therapeutic strategies have increasingly focused on macrophages as active targets rather than passive participants. Two conceptually distinct approaches have emerged: (i) macrophage-based cell therapy, involving the administration of ex vivo-programmed macrophages, and (ii) manipulation of host macrophages, aiming to reprogram endogenous macrophage populations in situ. Each strategy presents unique opportunities and challenges, particularly in the context of the cornea’s immune privilege and requirement for precise temporal control of inflammation.

### 8.1. Macrophage Cell Therapy: Opportunities and Limitations

Macrophage cell therapy involves the adoptive transfer of macrophages that have been preconditioned ex vivo toward anti-inflammatory or pro-resolving states. In principle, such macrophages could be engineered to promote resolution of inflammation, suppress myofibroblast persistence, and facilitate restoration of stromal architecture. Experimental studies in non-ocular tissues have shown that macrophages polarized toward resolution-associated or reparative programs can actively reverse fibrotic remodeling by clearing apoptotic cells, regulating ECM turnover, and suppressing profibrotic cytokine signaling.

Strategies that directly manipulate host macrophages have shown promise in reducing corneal fibrosis by shifting the wound microenvironment toward resolution. For example, pharmacologic modulation with an ATR inhibitor (AZD6738) attenuated macrophage infiltration and suppressed pro-inflammatory polarization through NF-κB signaling, leading to reduced inflammation and collagen deposition in an LPS-driven corneal injury model [94]. Similarly, MSC-derived EVs reduce fibrosis by decreasing pro-inflammatory cytokines and promoting an anti-inflammatory, M2-associated macrophage phenotype while also downregulating fibrotic gene expression (Col3a1, Acta2) [95]. In contrast to these host modulation approaches, macrophage cell therapy, such as adoptive transfer of ex vivo-polarized macrophages or engineered macrophage populations, has shown therapeutic potential in non-ocular settings and offers a conceptual framework for future corneal applications.

However, applying macrophage cell therapy to the cornea presents significant challenges. First, macrophage phenotypes are highly plastic, and ex vivo-programmed macrophages may rapidly lose their intended functional state once exposed to the inflammatory corneal microenvironment. Second, the introduction of exogenous immune cells into an immune-privileged tissue risks disrupting corneal homeostasis and triggering unintended inflammatory responses. Finally, defining a stable and universally beneficial macrophage “subtype” for transplantation remains difficult, as macrophage function is strongly dictated by timing, local cues, and injury context rather than fixed identity.

As such, while macrophage cell therapy provides valuable mechanistic insight and proof-of-concept, its translational applicability for corneal fibrosis remains uncertain.

#### Manipulating Host Macrophages: A Context-Dependent Strategy

In contrast, manipulation of endogenous macrophages seeks to reprogram resident and recruited macrophage populations within the cornea without introducing exogenous cells. This approach leverages the existing immune architecture of the tissue and allows macrophages to remain spatially and temporally integrated within the wound-healing cascade.

Strategies to manipulate host macrophages include modulation of cytokine signaling (e.g., IL-1, TGF-β, MIF), delivery of immunoregulatory factors, metabolic reprogramming, and use of cell-derived products such as MSC-derived EVs. Importantly, these approaches do not aim to suppress macrophage activity globally but instead promote the timely transition from inflammatory to resolution-phase programs, which is critical for preventing fibrosis while preserving early repair functions.

Evidence indicates that therapeutic manipulation of host macrophages is a feasible and effective strategy for limiting corneal fibrosis by restoring appropriate immune resolution rather than eliminating macrophages outright. In experimental models of corneal injury, pharmacologic and biologic interventions that modulate macrophage signaling pathways have been shown to reduce stromal scarring by suppressing prolonged inflammatory activation. For example, Xu et al. demonstrated that activation of the macrophage NLRP3 inflammasome amplifies epithelial TGF-β1 signaling and drives corneal fibrosis, while inhibition of this pathway attenuates myofibroblast persistence and stromal scarring [33]. Similarly, MSC-EVs have been shown to reprogram endogenous macrophages toward anti-inflammatory and pro-resolution states, resulting in decreased expression of fibrotic genes such as Acta2 and Col3a1 and reduced corneal opacity in alkali burn and inflammatory injury models [96]. Importantly, pharmacologic modulation of macrophage inflammatory signaling—for example, through suppression of NF-κB pathways—also reduces macrophage infiltration and inflammatory cytokine production, further limiting fibrosis without impairing early wound defense [94]. Collectively, these studies support host macrophage manipulation as a strategy that preserves essential early macrophage functions while promoting timely transition to resolution-phase programs critical for preventing corneal fibrosis.

In the cornea, where macrophages must coordinate epithelial repair, stromal remodeling, and immune privilege, host macrophage manipulation offers greater precision and safety than adoptive cell transfer. This strategy also accommodates the inherent heterogeneity of macrophage populations, allowing functional adaptation rather than enforcing a fixed phenotype.

### 8.2. Which Macrophage Subtype Should Be Targeted?

A central question in macrophage-based therapy for corneal fibrosis is which macrophage states should be promoted or suppressed. Accumulating evidence indicates that targeting broad categories such as “M1” or “M2” macrophages is insufficient and potentially misleading. Instead, therapeutically relevant macrophage programs are better defined by functional and temporal characteristics rather than rigid phenotypic labels.

Key regenerative macrophage states include resolution-phase macrophages, which suppress inflammation, clear apoptotic cells and excess ECM, and facilitate stromal normalization; anti-angiogenic macrophages, which preserve corneal avascularity and prevent vascular-driven fibrosis; and non-TGF-β-amplifying macrophages, which do not sustain myofibroblast survival or excessive matrix deposition. In contrast, macrophage states characterized by persistent inflammatory signaling, angiogenic activity, metabolic dysregulation, or failure of resolution represent critical targets for inhibition or therapeutic reprogramming. Importantly, these macrophage states are defined more by timing, signaling context, and microenvironmental cues than by canonical surface markers alone.

### 8.3. Conceptual Implications for Corneal Fibrosis Therapy

Collectively, current evidence favors reprogramming host macrophages toward resolution-associated states over macrophage cell replacement strategies. Rather than introducing a predefined macrophage subtype, effective therapy for corneal fibrosis likely requires restoring appropriate macrophage timing, ensuring early inflammatory functions occur but are efficiently terminated.

This perspective aligns closely with emerging regenerative approaches, including MSC- and EV-based therapies, which modulate macrophage behavior indirectly and dynamically. By shaping the macrophage microenvironment rather than replacing macrophages outright, such strategies may offer safer and more effective means of preventing or reversing corneal fibrosis.

## 9. Future Perspectives and Clinical Translation

Advances in our understanding of corneal immunobiology have reframed fibrosis not as an unavoidable consequence of injury, but as the result of misregulated immune dynamics, particularly involving macrophages. Translating these insights into clinical therapies requires a shift from static, cell-type-based interventions toward time- and context-dependent modulation of immune responses. This section outlines emerging strategies, biomarkers, and challenges that will shape the next generation of corneal fibrosis therapies.

### 9.1. Timing-Based Therapeutic Strategies

A central implication of macrophage-centric models of corneal fibrosis is that therapeutic efficacy depends critically on timing. Early inflammatory macrophage responses are essential for pathogen clearance, debris removal, and initiation of repair, and indiscriminate suppression at this stage can impair epithelial closure and stromal regeneration. In contrast, failure to terminate these inflammatory programs during the proliferative and remodeling phases promotes myofibroblast persistence, excessive ECM deposition, and stromal scarring.

Future therapeutic strategies should therefore aim to preserve early macrophage functions while accelerating or reinforcing resolution-phase programs. This may involve delayed intervention windows that promote macrophage reprogramming rather than early immunosuppression. Approaches such as MSC therapy, MSC-derived EVs, and targeted cytokine modulation are particularly well suited to this paradigm, as they can dynamically reshape macrophage behavior in response to the evolving wound microenvironment.

Clinically, this timing-based approach suggests that treatment regimens for corneal injury and surgery should be stratified by stage of wound healing, rather than applied uniformly. Defining optimal intervention windows remains a key translational challenge but also represents a major opportunity to prevent fibrosis without compromising essential immune defense.

### 9.2. Biomarkers of Macrophage Activation and Resolution

Successful clinical translation of macrophage-targeted therapies will require reliable biomarkers that distinguish regenerative from fibrotic immune trajectories. Traditional markers of inflammation provide limited insight into macrophage functional states and do not capture the dynamic transitions that determine healing outcomes.

Emerging candidates include cytokine profiles (e.g., IL-1β, TNF-α versus IL-10-associated signatures), pathway-specific markers such as inflammasome activation, and transcriptional programs identified through single-cell RNA sequencing. Markers reflecting macrophage origin (resident versus recruited), metabolic state, and interaction with stromal cells may further improve prognostic precision. Importantly, biomarker development should focus not only on macrophage activation but also on evidence of resolution, such as signals associated with myofibroblast clearance, ECM normalization, and restoration of corneal avascularity.

In the future, integrating tear-fluid analysis, imaging-based biomarkers, and minimally invasive molecular profiling could enable real-time monitoring of immune timing, allowing clinicians to adapt therapy before fibrosis becomes irreversible. Key therapeutic targets and macrophage-modulating strategies currently under investigation are summarized in Table 5.

### 9.3. Challenges and Open Questions

Despite rapid progress, several challenges must be addressed before macrophage-based strategies can be widely applied in the clinic. Patient heterogeneity represents a major obstacle, as age, systemic disease, prior inflammation, and genetic background can all influence macrophage behavior and wound-healing dynamics. Additionally, different corneal pathologies, such as chemical burns, infectious keratitis, surgical trauma, or neurotrophic disease, engage distinct immune programs, limiting the applicability of a one-size-fits-all approach.

Delivery methods also remain a critical consideration. Local versus subconjunctival administration, dosing frequency, and sustained delivery systems must be optimized to achieve sufficient immune modulation without disrupting corneal immune privilege. For cell-free approaches such as EV therapy, issues related to scalability, standardization, and cargo heterogeneity must be resolved to ensure reproducibility and regulatory approval.

Finally, fundamental questions remain regarding macrophage plasticity in vivo, including the stability of therapeutically induced states and the potential for maladaptive reprogramming under chronic inflammatory conditions. Addressing these gaps will require longitudinal studies and improved in vivo models that faithfully recapitulate human corneal disease.

### 9.4. Concluding Remarks

Collectively, the evidence reviewed here supports a paradigm shift in our understanding of corneal fibrosis, from a purely structural disorder to a consequence of failed immune timing. Macrophages emerge as central decision-makers in this process, capable of driving either regenerative healing or pathological scarring depending on when and how their programs are activated and resolved.

Rather than asking whether macrophages are beneficial or harmful, future therapies must ask when macrophage activity should be supported, redirected, or restrained. By embracing this temporal framework, macrophage-centered strategies, particularly those leveraging MSCs, EVs, and targeted immunomodulation, offer the potential to preserve corneal transparency, prevent fibrosis, and transform the clinical management of corneal disease.

## Figures and Tables

**Table 1:** Corneal Macrophage Heterogeneity Beyond the Traditional M1/M2 Classification: Representative Marker Genes and Putative Functions [51,57,58].

Macrophage Cluster	Top Marker Genes	Function
TLF^+^CCR2^−^ resident macrophages	Timd4, Lyve1, Folr2, Mrc1, Cd163, Igf1, Vegfb	Pro-resolving, phagocytic, tissue homeostasis, trophic support
TLF^−^CCR2^−^ resident macrophages	Ccl2, Ccl7, Hbegf, Egr2, Pf4	Mixed inflammatory-regulatory
TLF^−^CCR2^+^ resident macrophages	Ccr2, Cxcl16, H2-Ab1, H2-Eb1	Pro-inflammatory, antigen-presenting
CCR2^+^ macrophages	CCR2, Ly6C	Pro-inflammatory, injury-responsive
CCR2^−^ macrophages	CX3CR1, MHC-II-associated markers	Tissue surveillance, repair, immune regulation

Note: Longitudinal single-cell studies examining macrophage dynamics during corneal wound healing and fibrosis remain limited. Consequently, precise temporal abundance patterns for most macrophage subsets have not yet been defined, representing an important knowledge gap in the field.

**Table 2:** Temporal framework of macrophage activation during corneal wound healing. Phase-specific roles of macrophages across the inflammatory, transition/resolution, and late or persistent activation phases of corneal wound healing. Distinct macrophage functions, signaling cues, and biological outcomes dominate at each stage, with appropriate temporal progression supporting epithelial repair, stromal remodeling, and restoration of transparency. Dysregulation of macrophage timing, either insufficient early activation or failure to transition toward resolution, promotes sustained inflammation, myofibroblast persistence, ECM stiffening, and fibrotic scarring.

Wound- Healing Phase	Timing *	Dominant Macrophage Functions	Key Signals & Cues	Biological Outcomes	Consequences of Dysregulation
Early inflammatory phase	Hours to ~1–3 days	Debris clearance, pathogen defense, initiation of repair signaling	IL-1α/β, TNF-α, chemokines, epithelial damage signals	Epithelial migration, keratocyte apoptosis, provisional stromal remodeling	Insufficient activation → delayed wound closure; excessive activation → amplified inflammation
Transition/resolution phase	~3–7 days	Phenotypic reprogramming toward pro-resolution states, efferocytosis, immune regulation	Anti-inflammatory mediators, ECM cues, paracrine signals (including MSC-/EV-derived factors)	Suppression of inflammation, fibroblast apoptosis or reversion, restoration of stromal organization	Failed transition → persistence of inflammatory and profibrotic signaling
Late or persistent activation phase	≥7 days or chronic injury	Sustained profibrotic signaling, macrophage–fibroblast crosstalk	TGF-β, mechanotransduction signals, stiffened ECM	Myofibroblast persistence, excessive ECM deposition, increased stromal stiffness	Fibrotic scarring, stromal haze, irreversible loss of transparency

* Timing is approximate and varies with injury severity, disease context, and species.

**Table 3:** Functional heterogeneity of M2-associated macrophage subtypes relevant to corneal wound healing and fibrosis. M2-associated macrophages comprise a spectrum of functionally distinct programs that arise in response to specific cytokine and microenvironmental cues. These subtypes exhibit divergent roles in tissue repair, immune regulation, angiogenesis, efferocytosis, and fibrotic remodeling. In the cornea, M2-like macrophages can support epithelial regeneration, ECM remodeling, and immune resolution; however, sustained or dysregulated activation of certain M2-associated programs may contribute to pathological angiogenesis and stromal fibrosis. The functions listed represent dominant biological tendencies and may overlap or evolve dynamically depending on injury severity, local signaling, and temporal context.

M2-Subtype	Activating Stimulus (i)	Primary Function (s)	Key Associated Processes
M2a (Classical)	IL-4, IL-13	Wound healing, tissue repair, response to helminth infections, promotion of fibrosis.	Collagen synthesis, tissue remodeling, repair.
M2b (Immune Regulator)	Immune complexes, TLR agonists	Immune regulation, Th2 responses, production of both pro- and anti-inflammatory mediators.	B-cell activation, immune modulation.
M2c (Deactivation)	IL-10, TGF-β, glucocorticoids	Extracellular matrix remodeling, immunosuppression, deactivation of inflammation.	Tissue remodeling, immune suppression.
M2d (Pathological Angiogenic)	TLR ligands (e.g., LPS), Adenosine A2a receptor agonists	Promotes angiogenesis (formation of new blood vessels), tumor progression.	New blood vessel formation.
M2eff (Efferocytic)	Phagocytosis of apoptotic cells (efferocytosis)	Anti-inflammatory response to clear dead cells, production of IL-10 and TGF-β.	Clearing cellular debris post-inflammation.

**Table 4:** Macrophage transcriptional states identified in corneal single-cell transcriptomic studies. Single-cell RNA sequencing analyses have revealed that corneal macrophages comprise multiple transcriptionally and functionally distinct populations rather than discrete M1/M2 subsets. These states reflect dynamic programs associated with inflammation, antiviral defense, antigen presentation, immune resolution, fibrosis, metabolic adaptation, and tissue homeostasis. The markers and putative functions listed represent dominant transcriptional signatures reported across corneal and ocular surface single-cell studies and are influenced by injury severity, disease context, and temporal stage of wound healing.

Macrophage Transcriptional State	Representative Markers	Putative Functions in Cornea
Inflammatory monocyte-derived macrophages	Lyz2, Ccr2, Il1b, Tnf, S100a8/a9	Early injury response, cytokine production, immune cell recruitment, keratocyte apoptosis
Interferon-responsive macrophages	Ifit1, Ifit3, Isg15, Stat1	Antiviral defense, amplification of innate immune signaling, inflammatory persistence in severe injury
Antigen-presenting/immune-interacting macrophages	H2-Ab1, Cd74, Mhc II genes	Crosstalk with adaptive immune cells, immune surveillance, modulation of chronic inflammation
Pro-resolution/reparative macrophages	Mrc1, Cd163, Il10, Retnla	Efferocytosis, inflammation resolution, support of epithelial repair and stromal remodeling
Profibrotic macrophages	Tgfb1, Spp1, Fn1, Ctgf	Myofibroblast activation, ECM deposition, promotion of stromal fibrosis
Tissue-resident-like macrophages	Cx3cr1, F4/80, low Ccr2	Homeostatic surveillance, regulation of immune privilege, modulation of injury responses
Metabolically activated macrophages	Fabp5, Lpl, Apoe	Lipid handling, regulation of chronic inflammation, potential contribution to fibrotic persistence

**Table 5:** Therapeutic targets and macrophage-modulating strategies in corneal fibrosis. Summary of molecular pathways and pharmacologic or biologic interventions targeting macrophage activation and fibrotic signaling in corneal wound healing. Interventions that restore phase-appropriate macrophage function, reducing IL-1β/TGF-β-driven inflammation while enhancing resolution and matrix remodeling, hold promise for scar-free regeneration.

Target	Therapeutic Approach	Outcome
IL-1β/NLRP3	MCC950, IL-1R antagonists	↓ Inflammation ↓ TGF-β ↓ Fibrosis
TGF-β1/ALK5	Partial inhibitors	↓ Myofibroblast persistence
CCR2/CCL2 axis	Recruitment blockers	↓ Macrophage infiltration
MMP12	Enzyme enhancers/mimetics	↓ Fibrosis ↓ Angiogenesis
Polarization	IL-10, IL-4, resolvins, MSC therapy	Promote resolution ↓ Scarring
PEDF from cMSCs	cMSC transplant or mimetics	Anti-angiogenic Anti-fibrotic
Transcriptomic targeting	Subset-specific gene modulation	Precision therapy

↓ indicates a decrease or reduction in the indicated biological process.

## Data Availability

The original contributions presented in this study are included in the article. Further inquiries can be directed to the corresponding author.

## Abbreviations

The following abbreviations are used in this manuscript:

ECM	Extracellular matrix
EVs	Extracellular vesicles
LSCs	Limbal stem cells
MSCs	Mesenchymal stromal cells
IL	Interleukin
MMP	Matrix metalloproteinase
TGF-β	Transforming growth factor beta
TNF-α	Tumor necrosis factor alpha
VEGF	Vascular endothelial growth factor
IL-1α	Interleukin-1 alpha
IL-1β	Interleukin-1 beta
α7nAChR	Alpha-7 nicotinic acetylcholine receptor
ALK5	Activin receptor-like kinase 5
ARG1	Arginase 1
cMSCs	Cornea-derived mesenchymal stromal cells
CCL1	C-C motif chemokine ligand 1
CCR2	C-C chemokine receptor 2
CXCL8	C-X-C motif chemokine ligand 8
F4/80	EGF-like module-containing mucin-like hormone receptor-like 1 (EMR1)
Fizz1	Found in inflammatory zone 1
LPS	Lipopolysaccharide
MIF	Macrophage migration inhibitory factor
Mrc1	Mannose receptor C-type 1
MCC950	NLRP3 inflammasome inhibitor (MCC950)
Mgl1	Macrophage galactose-type lectin 1
Mgl2	Macrophage galactose-type lectin 2
NF-κB	Nuclear factor kappa B
NLRP3	NLR family pyrin domain containing 3
PDGF	Platelet-derived growth factor
PEDF	Pigment epithelium-derived factor
qPCR	Quantitative polymerase chain reaction
STAT3	Signal transducer and activator of transcription 3
Th1	T helper 1
Th2	T helper 2
TLR	Toll-like receptor
UMSCs	Umbilical cord-derived mesenchymal stromal cells
Ym1	Chitinase-like protein 3
